# Exposure of a single wild boar population in North Rhine-Westphalia (Germany) to perfluoroalkyl acids

**DOI:** 10.1007/s11356-022-23086-6

**Published:** 2022-09-28

**Authors:** Carsten Felder, Lukas Trompeter, Dirk Skutlarek, Harald Färber, Nico Tom Mutters, Céline Heinemann

**Affiliations:** 1 Institute for Hygiene and Public Health, University Hospital Bonn, Medical Faculty, University of Bonn, Building 63, Venusberg-Campus 1, 53127 Bonn, Germany; 2 Institute of Animal Science, University of Bonn, Katzenburgweg 7-9, 53115 Bonn, Germany

**Keywords:** PFAA, PFAS, Liver, Wild boar, Environmental pollution, Poly- and perfluorinated substances, Risk assessment

## Abstract

Perfluoroalkyl acids (PFAA) are among the leading chemical pollutants in the twenty-first century. Of these, perfluorooctanoic acid (PFOA) and perfluorooctanesulfonic acid (PFOS) have been widely detected in a large number of animal and environmental samples. Wild boars accumulate PFAA in their livers, but it has not yet been clarified to what extent wild boars of the same population accumulate different PFAA in their livers or whether any conclusions can be drawn from any differences found in regard to environmental contamination. In this study, liver samples from wild boars killed during driven hunts in 2019 and 2020 from a defined forest area in North Rhine-Westfalia, Germany were analyzed for 13 different PFAA. A mean load of 493 µg/kg (± 168 µg/kg) PFAA was measured in 2020. Perfluorosulfonic acids accounted for 87% of the total load in both years, with PFOS dominating this group. These results were similar to those of 14 liver samples collected from other regions of Germany for comparison. In addition, the livers of hunted pregnant sows and fetuses were examined. The load of short-chain perfluorocarboxylic acids (< C8) in the fetus liver was as high as that of the sows, whereas the concentrations of long-chain perfluorocarboxylic acids (≥ C8) were lower than in the dams. This result shows for the first time that fetuses take up PFAA from their mothers in utero. Our study shows that PFAA content in wild boar livers is comparably high in all animals in a local population and indicates a need for further research regarding a nationwide background exposure to PFAA in wild boars and their surrounding environment.

**Supplementary Information:**

The online version contains supplementary material available at 10.1007/s11356-022-23086-6.

## Introduction

Per- and polyfluorinated alkyl substances (PFAS) are anthropogenic chemicals used in many industrial sectors since their discovery in the 1950s due to their water, oil, and dirt-repellent properties (Buck et al. 2011). PFAS are used in many applications, such as surface treatment in the textile and paper industries, galvanic processes, non-stick consumer goods, electronic devices, cosmetics and household items, or as an ingredient in the formulation of fire extinguishing agents (Fujii et al. 2013; Høisæter et al. 2019; Silva et al. 2021). There are now over 4700 known PFAS with fully or partially fluorinated alkyl chains (Glüge et al. 2020). Due to the stability of these C-F bonds, PFASs are extremely stable against photochemical, oxidative, and microbial degradation. In this context, perfluoroalkylcarboxylic acids (PFCA) and perfluoroalkylsulfonic acids (PFSA), which form the group of PFAA, are the terminal degradation products found in the environment (Cousins et al. 2020).

PFAA are divided into a short-chain (< C8) and a long-chain group (≥ C8), depending on each substances half-life in humans, which increases significantly above a C8 carbon chain. The lead substances of the long-chain groups are perfluorooctanoic acid (PFOA) and perfluorooctanesulfonic acid (PFOS). The half-life for short-chain PFAA in the human body is a few days to months, whereas PFOA and PFOS remain in the human body for several years (Cousins et al. 2020). Specifically, PFOA and PFOS are absorbed in the gastrointestinal tract after ingestion, then bind to nonspecific serum albumin in the blood (Han et al. 2003). Once in the blood, they distribute throughout the organism, finally accumulating in well-vascularized organs, such as the liver and kidneys (Pérez et al.^2013^). PFAA are associated with a couple of health risks. Serum concentrations of PFOA and PFOS are associated with an increased level of total cholesterol (Dong et al. 2019). Furthermore, PFOA, PFOS, perfluorononanic acid (PFNA), and perfluorohexanesulfonic acid (PFHxS) in children are showing an inverse association with antibody titers against diphtheria, tetanus, and haemophilus influenza type b (Hib b) (Grandjean et al. 2017; Abraham et al. 2020). PFOA and PFOS can also pass into the bloodstream of fetuses via the umbilical cord and accumulate in fetal organs (Fromme et al. 2010; Mamsen et al. 2019). Various biomonitoring programs have also detected PFAS in human blood (Olsen et al. 2017; Pirard et al. 2020; Göckener et al. 2020). In humans, these substances are taken in primarily via food and, to a lesser extent, contaminated dusts in indoor air (German Federal Institute for Risk Assessment 2021).

Since not only PFAA, but all PFAS have no natural origin, the starting point for their distribution in the environment is always anthropogenic. PFAS sources can be a result of their intended use, for example as a component of firefighting foams or from unintentional use, such as during the use of contaminated sewage sludge as a soil conditioner (Høisæter et al. 2019; Röhler et al. 2021). PFAS can disperse in the environment via transport pathways in water and soil matrices or via the air, thus contaminating large areas, which has even resulted in their ubiquitous detection far from human civilization (Galatius et al. 2013; Skutlarek et al. 2006). It follows that a background level of contamination now exists on Earth at levels that vary depending on a number of factors.

The uptake and accumulation of PFAS in flora and fauna depends on the background pollution level, as well as the metabolic pathways and dietary habits of each species. For example, PFOS has been measured in cod, mink, and polar bear livers; the results of these studies show that PFOS concentrations are higher in predators than prey (Boisvert et al. 2019; Smithwick et al. 2005; Valdersnes et al. 2017; Persson et al. 2013). High levels of PFAS (primarily PFOA and PFOS) have also been detected in wild boar (*Sus scrofa*) livers. Wild boars are omnivores that spend a lot of time digging through the soil for food. In doing so, the animals ingest fungi, small animals, insects, and plant matter, such as roots and tubers, as a part of their diet, all of which can accumulate PFAS, even at low levels (Kowalczyk et al. 2018; Falk et al. 2019).

Wild game meat is considered a natural and sustainable meat source by consumers because the animals do not come from conventional animal husbandry, are able to act out their natural behaviors, and are raised without any pharmaceuticals (Demartini et al. 2018). In Germany, wild boar meat is playing an increasing role in food production. In addition to its increased popularity among consumers, hunting of wild boar has increased to combat African swine fever, which has led to an accompanying increase in supply (LAVES 2021). In Germany, the number of wild boars killed has doubled from the 2009/2010 hunting season to the 2019/2020 season (440,354 to 882,231 individuals) (Deutscher Jagdverband 2021). Investigations regarding the amount of PFAA contamination in wild boar play an increasing role in preventive consumer protection given this increase in wild boar supply and contamination.

The livers and muscle meat of over 500 wild boars shot in Hesse between 2007 and 2009 were investigated for PFOA and PFOS contamination in an extensive 2012 study. The mean and median PFOS values measured in the livers were 117 µg/kg and 49 µg/kg, respectively, whereas both values were below the limit of quantification for PFOA (Stahl et al. 2012). Later, Kowalczyk et al. (2018) summarized the data published for wild boar livers from Germany and found that the mean PFOS values ranged from 117 µg/kg in Hesse to 407.8 µg/kg in Bavaria (Kowalczyk et al. 2018). In northern Italy, wild boar livers had a mean PFOS load of 150 µg/kg (Barola et al. 2020). The samples examined in these studies were random samples provided to the study authors by hunters from large areas. Based on these studies, it is possible, especially with such a large number of samples as in Stahl et al. (2012), to determine the superregional load; however, it is difficult to draw conclusions regarding the load’s distribution in a local population from this data, as it remains unclear if one liver sample can be seen as representative for the entire local population. Thus, the aim of this study was to analyze the PFAA exposure of wild boars in a single population in more detail. Specifically, a detailed analysis spectrum of PFCA and PFSA was performed on a large number of samples from a small geographical area to determine how strongly PFAA loads in wild boar livers differ within a single population and to investigate whether a load pattern could be identified in these animals. Additionally, fetus livers from this population were investigated to determine whether fetuses can accumulate PFAA in utero from their dams.

## Material and methods

### Chemicals and materials

A PFAA mixed standard (Wellington Laboratories Inc., Guelph, Canada) was used for quantification. This contained perfluorobutanoic acid (PFBA), perfluoropentanoic acid (PFPeA) perfluorohexanoic acid (PFHxA), perfluoroheptanoic acid (PFHpA), PFOA, PFNA, perfluorodecanoic acid (PFDA), perfluorundecanoic acid (PFUnDA), perfluorododecanoic acid (PFDoDA), perfluorobutanesulfonic acid (PFBS), PFHxS, perfluoroheptanesulfonic acid (PFHpS), and PFOS. A monoisotopically labeled mixed standard (Wellington Laboratories Inc., Guelph, Kanada) consisting of 13C-PFBA, 13C-PFHxA, 13C-PFOA, 13C-PFNA, 13C-PFDA, 13C-PFUnDA, 18O-PFHxS, and 13C-PFOS was used as an internal standard. All other chemicals used were purchased from Carl Roth GmbH + Co. KG (Karlsruhe, Germany) and VWR International GmbH (Darmstadt, Germany) and met grade requirements for high-performance liquid chromatography—mass spectrometry.

### Study area

A 22-km^2^ area of a forest south of Bonn, Germany, (measured via timm-online.nrw.de) was investigated in this study. The study site is bordered on the south and west by large roads with fences bordering the forest to ensure that wild animals do not cross the roads. To the north and east, the forest area borders Bonn. According to the local district forester, the wild boars in this area are not known to leave this forest area, as it has an abundant food supply. The study area is part of a larger forest name the Kottenforst, which forms the southwestern end of Rhineland Nature Park, a 1045-km^2^ nature park in southern North Rhine-Westphalia (Naturpark Rheinland). The forest area has been designated as a nature reserve since the middle of the twentieth century and was historically used for forestry. In the past, no soil conditioners or similar substances that could cause pollution were applied. The Forest Service is not aware of any large fires or associated firefighting activities in the forest’s vicinity that could have resulted in PFAS exposure from firefighting foams. Despite these facts, the forest area is surrounded by urban areas. To the west are, among others, a federal road and a highway, whose rain runoff enters the Kottenforst via a system of historic drainage ditches. The water surfaces in the forest area are fed exclusively by rainwater, since the groundwater lies below an impermeable rock layer of Rhine gravel and pseudogley. For this reason, rainwater forms small streams that drain northward toward the city.

### Sample collection

In December 2019 and November and December 2020, a total of 90 samples (68 in 2020) were collected from the livers of hunted wild boars during three driven hunts. During the 2020 hunts, 57 muscle samples were also collected from the foreleg of the animals. Fetus livers from the pregnant, hunted sows were also sampled. All samples were collected immediately following the hunts during evisceration. At least 50 g of sample material per animal was packed in polyethylene bags and kept refrigerated during transport. In addition, the sex of the animals and a classification of body weight into < 20 kg body weight (BW) (juveniles) and ≥ 20 kg BW (adults) groups were recorded as part of the mandatory Trichinella monitoring process. The fetuses from four pregnant sows hunted in 2020 were able to be entirely removed. Following the hunt, the fetuses were dissected, and their complete livers were taken as samples. For three sows all livers of a litter were pooled together for technical reasons, because the sample size of each fetus alone was too small. In one sow, physical development of the four fetuses had progressed to the point that the livers could be used as individual samples. All samples were frozen the same day at − 40 °C and stored until analysis. The information on sex, weight group, and the year in which they were shot are given for each animal in Table S1.

In spring 2021, additional 14 liver samples were collected from wild boars in Hesse, Rhineland-Palatinate, and the Eifel region. These samples were provided to the laboratory by hunters or were purchased at a regional game trade. Of these, eight samples were taken from wild boar in Hesse to serve as a control group to the wild boars from this study. For seven samples out of this group, the hunted wild boars were weighed before evisceration and the animals were divided by the hunters into two age groups (< 1 year and 1–2 years), for one sample this data was not submitted by the hunter. For the other six samples (three each from Rhineland Palatinate and the Eifel region), age and bodyweight was not documented by the game traders, as this data is not necessary for selling the liver as grocery. These samples were also used in the control group and represent livers which were sold for food consumption.

### Sample preparation

The collected samples (liver and muscle tissue) were ground using a kitchen blender (Caso Design –Arnsberg, Germany). Then, 1 g was weighed into a polypropylene tube to which 20 mL methanol was added. The samples were then dispersed using an Ultra Turrax disperser (IKA –Staufen, Germany) for 2.5 min at 10,000 rpm and left overnight at room temperature. After extraction, purification via solid phase extraction was performed using Chromabond HR-X cartridges (Macherey & Nagel-Düren, Germany) according to DIN 38,407–42. The cartridges were each conditioned with 2 ml methanol + 0.1% formic acid, methanol, and water. Subsequently, 1 mL of the sample extract was mixed with 1 mL water + 0.1% formic acid and 50 µL internal standard (0.02 ppm) in the cartridge. After sample addition, the cartridges were rinsed with 2 mL of water + 0.1% formic acid, acetone:acetonirile (1:1) + 1% formic acid, and methanol. The samples were eluted using 3 ml methanol containing 0.1% NH_3_. The eluate was evaporated to dryness and reconstituted with 1 mL methanol:water (4:6) + 0.02% NH_3_. This was followed by measurement via high-performance liquid chromatography- tandem mass spectrometry (HPLC–MS/MS).

### Instrumental analysis

#### Chromatography

For chromatographic separation, an Agilent 1290 Inifnity II system (Agilent Technologies GmbH, Waldbronn, Germany) equipped with a ZORBAX Eclipse Plus C18 2.1 × 50 mm 1.8 Micron column (Agilent Technologies GmbH, Waldbronn, Germany) and a ZORBAX Eclipse Plus C18 2.1 × 5 mm 1.8 Micron Guard column, tempered to 50 °C in a column oven was used. The injection volume was 20 µl. To prevent carryover between the samples, the Agilent system’s multi-wash function was used, which involved rinsing the sample needle and needle seat sequentially with isopropanol + 0.1% formic acid, acetonitrile + 0.1% formic acid, and water + 0.1% formic acid for 3 s each. For the mobile phase, H_2_O containing 20 mM ammonium acetate and 2% acetonitrile (A) and methanol containing 20 mM ammonium acetate (B) were used at a flow rate of 0.5 ml/min. The gradient is shown in Table S2.

#### Mass spectrometry

Mass spectrometric measurements were performed on an Agilent Ultivo system (Agilent Technologies GmbH, Waldbronn, Germany) with electrospray ionization performed in negative mode. Detection was performed in dynamic multi-reaction-monitoring mode, with two characteristic mass transitions for each analyte. Exceptions were PFBA and PFPeA; one characteristic transition was used for each of these. Due to the short length of the carbon chain, only one characteristic transition could be identified with a sufficiently strong response. The mass transitions with retention time for all analytes and internal standards are listed in Table S3. The Agilent MassHunter workstation (Aquisition V 1.1 and Quant V 10.0; Agilent Technologies GmbH, Waldbronn, Germany) was used for instrument operation and quantitative evaluation. The Limits of Quantification for each individual PFAA ranged between 0.01 µg/kg (PFHxA) and 0.32 µg/kg (PFHpS). Recovery rates ranged between 95% (PFHpA) and 115% (PFBS). Detailed data for both limits of detection and quantification and recovery rates are given in the Tables S4 and S5.

#### Data analysis

For statistical analysis, we used IBM SPSS Statistics 28 (IBM, Bonn, Germany). Students’ *t*-test was performed to compare samples from the two years and sex differences. A significance level of 0.05 was selected.

## Results and discussion

**Fig. 1:**
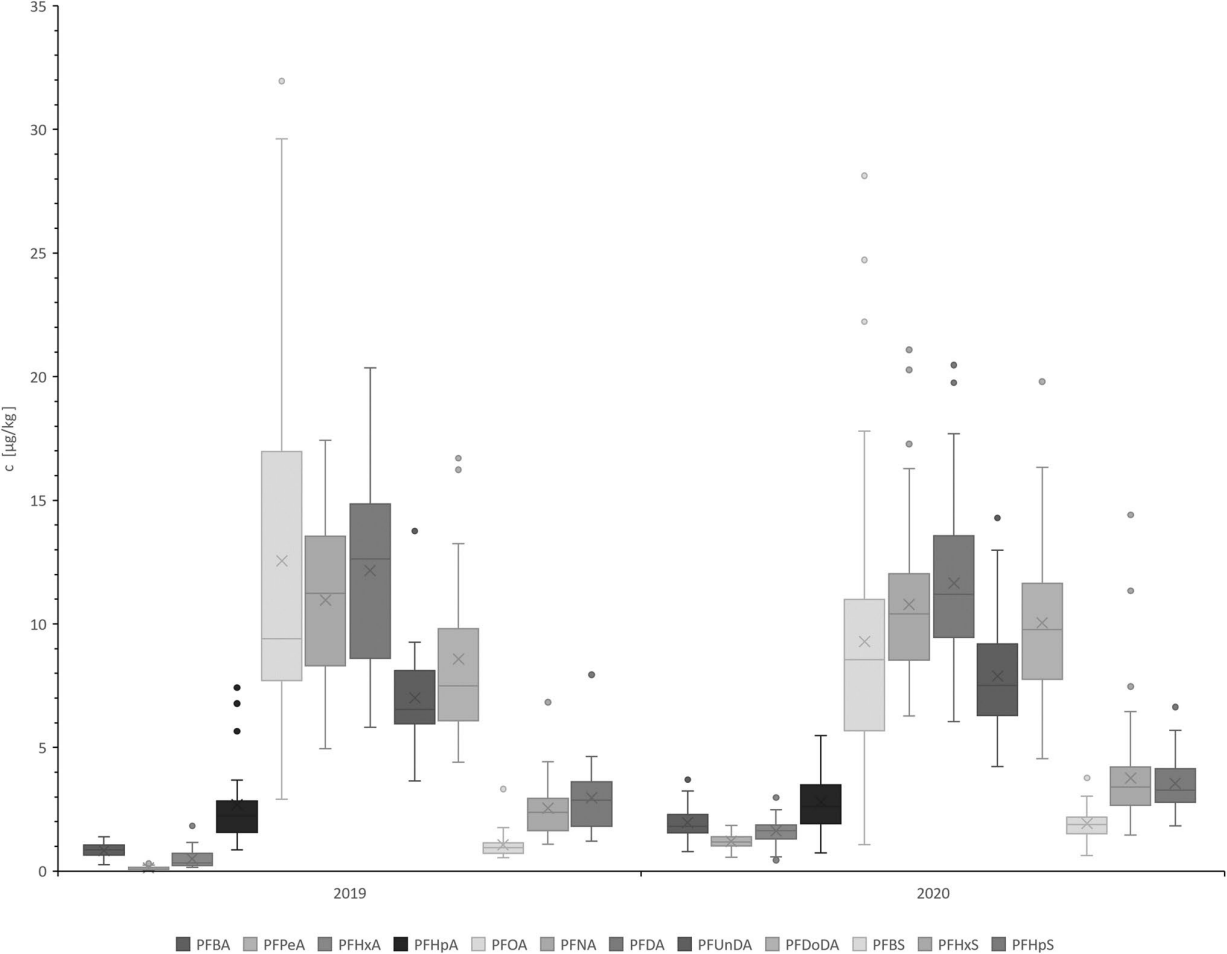
Distribution of PFAA load in wild boar livers from a geographically delimited area in 2019 and 2020. The distributions for the ≥ 20 kg BW group clearly show that the concentrations of the studied PFAS (except PFBA to PFHxA) are all in the same order of magnitude, despite differing mean values

Table 1 shows the PFAA values measured in this study, grouped by year and weight class for the Kottenforst study area, and the result for the individual animals is given in Table S6. Results for the samples from the other geographical areas are given in Table S7. For the Kottenforst- study area, PFOS dominates among all analytes in terms of quantity. The mean value for PFOS of the ≥ 20 kg BW group was constant over the two years, whereas the mean value of the < 20 kg BW group increased significantly (*p* = 0.01). In 2019, the livers of only five animals with a bodyweight of less than 20 kg were examined, while 20 samples from this group were measured in 2020, making the values difficult to compare. Over the totality of all samples tested from the Kottenforst study area, PFOS contributed 85.9% ± 4.1% of the total PFAA load. When comparing the PFAA load of female and male animals, there were no significant differences in the < 20 kgBW group. In the ≥ 20 kgBW group only PFOA showed a significant difference (*p* ≤ 0.01), with mean values of 7.6 µg/kg ± 4.3 µg/kg (female) and 15 µg/kg ± 7.9 (male). The adult animals also showed little difference in the levels of PFHpA and long-chain carboxylic acids. The levels of the remaining short-chain PFAA (both carboxylic and sulfonic acids) increased in both groups from 2019 to 2020 (Fig. 1). As the length of the fluorinated alkyl chain increases, its half-life in the organism increases (Buck et al. 2011). Therefore, exposure to long-chain PFAA is more stable and increased over time, because these substances are absorbed more rapidly than they can be excreted, which causes their accumulation over the lifetime of an animal (Boisvert et al. 2019; Cousins et al. 2020). Whether a temporal trend can be detected in the dietary intake of short-chain PFAA cannot be determined using data from two consecutive years only; such investigation requires further research.

**Table 1:** PFAA concentration values (medians) in wild boar liver samples from a single German population in 2019 and 2020, differentiated into young animals (< 20 kg BW) and adults (≥ 20 kg BW) in µg/kg

	2019				2020			
Substance	< 20 kgBW *n* = 5		> 20kgbw *n* = 17		< 20 kgbw *n* = 20		> 20kgbw *n* = 48	
PFBA	0.81	(0.83)	0.84	(0.87)	2.1	(2.0)	1.9	(1.8)
PFPeA	0.06	(0.06)	0.15	(0.12)	1.7	(1.1)	1.2	(1.2)
PFHxA	0.29	(0.32)	0.56	(0.37)	1.6	(1.6)	1.6	(1.6)
PFHpA	2.0	(2.0)	2.9	(2.4)	2.6	(2.7)	2.8	(2.6)
PFOA	8.9	(7.8)	14	(12)	8.0	(8.7)	9.8	(8.5)
PFNA	8.4	(8.4)	12	(12)	11	(11)	11	(10)
PFDA	8.0	(7.5)	14	(14)	11	(11)	12	(11)
PFUnDA	5.0	(4.6)	7.6	(7.)	8.0	(7.8)	7.8	(7.2)
PFDoDA	5.8	(5.6)	9.4	(8.3)	11	(10)	9.8	(9.6)
PFBS	0.90	(0.9)	1.1	(0.99)	2.1	(2.0)	1.9	(1.8)
PFHxS	1.7	(1.6)	2.8	(2.6)	3.3	(3.3)	4.0	(3.5)
PFHpS	1,6	(1,6)	3,4	(3,2)	3,3	(3,3)	3,7	(3,4)
PFOS	200	(210)	460	(460)	340	(328,77)	460	(440)

In the case of long-chain PFAA, there were always outliers in the upper quartile, especially in 2020. This could be because the ≥ 20 kg BW covers a wide age and weight range of the animals, since wild boar life expectancy is 7–8 years with weights of up to 150 kg in Germany(Deutscher Jagverband, n. d). The livers obtained from eight wild boars from Hesse in spring 2021 showed a comparable range of PFAA concentrations to those in samples obtained from the Kottenforst study area. When considering the PFOS contents of the liver from the state of Hesse in relation to the wild boars’ BW (Table 2), it appears that BW and age are not the only factors influencing PFOS exposure. For example, liver samples from two animals of the same weight (31 kg) showed PFOS content of 534.36 µg/kg and 288.98 µg/kg, respectively, despite both animals being classified as under 1 year of age. This high PFOS level is more than twice the median value of this group, and is in the upper 25% quartile of all animals in the Kottenforst area. Thus, this result does not seem representative of the general trend in PFOS content levels in wild boar livers from this study.

**Table 2:** Comparison of body weight (BW) and PFOS load in wild boar livers

Animal No	Age	Body weight [kg]	PFOS [µg/kg]
1	1–2 years	49	160
2	< 1 year	40	200
3	< 1 year	35	250
4	< 1 year	31	530
5	1–2 years	35	260
6	1–2 years	32	200
7	< 1 year	31	290

The uptake of PFAA is mainly via diet, which could explain why some liver samples show significantly higher values, as the diet consumed by each individual varies, which results PFAA loads that are not homogeneous. There could be components in wild boar diets that are particularly highly contaminated, but it is not yet clear whether this high load is from a specific diet component or local/temporal variation. Load could increase sharply in animals that have recently ingested highly contaminated feed, but this high load can be balanced out again in a growing animal as the weight of the liver increases. On average, PFOS accounted for 85.9% ± 4.0% of the total PFAA load in the wild boars from the Kottenforst. In 2019, the proportion was 86.0% ± 3.4%, while it was 85.9% ± 4.0% in 2020. For the 13 samples obtained from other regions in spring 2021, the proportion was 85.1% ± 3.1%. The mean values of the individual regions varied between 84.9 and 86.5%.

### PFAA load in livers of wild boar fetuses

**Fig. 2:**
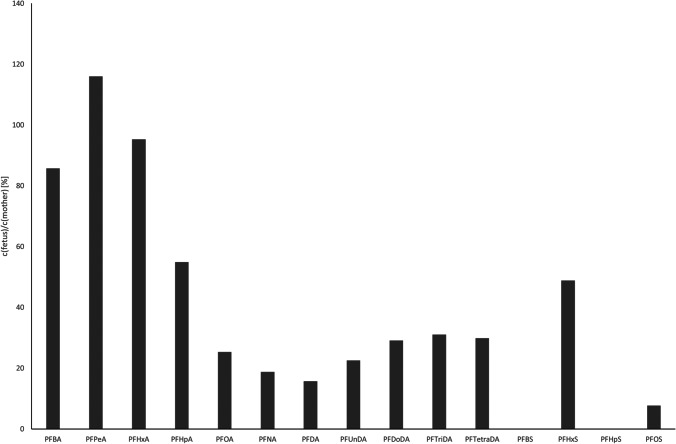
Relationship of PFAS concentrations in livers of fetal wild boars to their dams

The sum of the PFAA concentrations in pooled liver samples of wild boar fetuses ranged between 29 and 57 µg/kg, and the concentrations for all substances are given in Table 3. As also described above for the liver samples of the wild boars, PFOS showed the highest concentrations among all PFAA detected in the livers of the wild boar fetuses. All analyzed PFCA could be quantified in all samples with concentrations ranging between 1.0 and 4.6 µg/kg. Except for PFOS, the only quantifiable PFSA was PFHxS in one sample, with a concentration of 1.3 µg/kg. PFOS contributed with 67.7% ± 7.1% to the total PFAA load of the fetal livers. This proportion is significantly smaller than in the samples of the dam’s livers (*p* = 0.02), where PFOS accounted for 87.6. % ± 2.0% of the PFAA load. Figure 2 shows the ratio of the concentrations in the livers of the fetuses to those in the livers of their mothers. The highest ratio was found for PFPeA, with concentrations of 119% ± 13% of what was found in the mothers’ livers. PFOS showed the lowest ratio of 6.8% ± 1.9%.

**Table 3:** PFAS concentrations in pooled liver samples from wild boar fetuses in µg/kg

	Fetuses of sow no			
Analyte	2020–52	2020–31	2020–60	2020–65
PFBA	1,1	1,2	1,0	1,4
PFPeA	1,2	1,5	1,1	1,2
PFHxA	1,2	1,3	1,0	1,7
PFHpA	1,3	1,8	1,3	1,1
PFOA	1,9	4,6	1,8	2,0
PFNA	1,6	2,7	1,4	1,7
PFDA	1,5	1,7	1,3	1,8
PFUnDA	1,6	1,7	1,4	1,5
PFDoDA	1,9	2,	1,9	2,2
PFBS	< LOD	< LOD	< LOD	< LOD
PFHxS	< LOD	< LOD	< LOD	1,3
PFHpS	< LOD	< LOD	< LOD	< LOD
PFOS	37	38	17	41

Looking into PFCA, it seems like there is a negative correlation between the length of the carbon chain and the ratio between concentrations in fetal livers and the mothers’ liver between PFPeA and PFDA. Increasing length of the carbon chain is associated with decreasing mobility and increasing half-life in organisms (Knutsen et al. 2018).

To our knowledge, this is the first study to show fetal liver exposure to PFAA. The implications of this and also a possible inference of what this means for human fetuses are difficult to assess, but this result could be a cautionary sign. In humans, PFAA are associated with a number of negative health traits. For newborns, for example, there is evidence of reduced birth weight and negative effects on the immune system (Dalsager et al. 2016; Impinen et al. 2019; Meng et al. 2018). Whether these aspects also affect wild boar cannot be said at this time.

### Muscle meat

Only PFOS could be quantitatively detected in the muscle samples tested, with a detection ratio of 95%. The concentrations for all samples are given in Table S8. The mean value was 8.1 µg/kg ± 3.5 µg/kg. If the ratio between the PFAA determined in the liver samples is taken as a basis, the concentrations of the other PFAA to be expected in the muscle samples were below the detection limit, which is a logical explanation for our results. The muscle samples were also more inhomogeneous in composition compared to the liver samples. While the obtained liver tissue consisted largely of uniform liver cells, the obtained muscle samples contained fat, connective tissue, and tendons in addition to muscle fibers. In order to represent practical conditions, the samples were comminuted as a whole and then analyzed as an aliquot. This non-uniform composition of the different muscle samples can increase scatter, depending on each animal’s fat content. The PFOS concentrations in the muscle samples corresponded to 2.1% ± 1.3% of the PFOS concentrations in the liver samples. These results agree with those of Stahl et al. (2012), who found that the PFOS concentrations in muscle samples corresponded to 2.8% of the liver concentrations.

### Wild boar as food producing animal

In the fall of 2020, the value of the tolerable weekly intake (TWI) for the sum of PFOA, PFNA, PFHxS, and PFOS was lowered to 4.4 ng/kgBW per week by the European Food Safety Authority (EFSA) (Schrenk et al. 2020). Thus, for a 70-kg BW human, a weekly intake of 308 ng of these PFAA is tolerable. The median sum of the four PFAA selected by EFSA was 408.59 µg/kg for the entirety of the wild boar liver samples examined in 2020, thus a weekly consumption of 0.75 g of wild boar liver would be acceptable for humans. In the muscle meat samples examined in this study, only the PFOS concentration was above the detection limit. Even if only PFOS in the muscle meat is apportioned to the TWI, a median of 7.29 µg/kg means that a weekly intake of 42.25 g of muscle meat is tolerable, although this consideration neglects the fact that other foods may also be contaminated with PFAA (Knutsen et al. 2018). Against this background, food containing wild boar liver cannot be considered safe. For this reason, the state of Hesse banned the sale of wild boar liver at the beginning of 2021. The sale of muscle meat is still permitted, partly because of the lower concentrations and the fact that boar meat is not a regular part of diets in this region. In the rest of Germany, the sale of wild boar liver as a food product continues to be permitted. In three livers purchased from a regional supplier, the sum of the PFAA concentrations evaluated by EFSA ranged from 305 to 495 µg/kg. These values are within the range of mean value variation of the samples from the Kottenforst investigation area. Considering all the data, it seems likely that wild boar liver in western Germany contains PFAA to an extent that it cannot be considered a safe food.

### Consideration of the relationship of the PFAA

Due to the area-wide contamination of the examined wild boar livers with PFAA, the question arises whether a contamination pattern can be identified on the basis of the ratio of the individual substances to each other, which can be considered as background contamination. If such a pattern could be identified, a deviation from this pattern could serve as an indicator for a local contamination site. To test this hypothesis, the contribution of each analyte to the total sum of PFAA in the liver samples was considered. A prerequisite for the identification of an incident by a deviation from the usual substance pattern would be that the load is so homogeneously distributed within the population that a fluctuation represents a peculiarity.

The total of the analyzed PFCA corresponded to 14% ± 4% of the overall load in both years for samples out of the Kottenforst study area. Within PFCA, the proportions of the individual substances varied greatly. Especially for the short-chain analytes, the standard deviation was as high as the mean values. The reason for this is their short half-life in animals. For example, PFBA is excreted only a few days after ingestion, which is why the concentration in the liver is strongly dependent on the diet of the last few days (Knutsen et al. 2018). Therefore, to answer the question of whether there is a stable distribution pattern, only the long-chain PFCAs were considered. Table 4 shows the ratios of the long-chain carboxylic acids of the liver samples from both years, which was stable over the two years; however, the standard deviation was higher for PFOA than for the other PFCA.

**Table 4:** Percentage distribution of long-chain PFCA levels in wild boar livers

Year	PFOA [%]	PFNA [%]	PFDA [%]	PFUnDA [%]	PFDoDA [%]
2019	23.4 ± 7.4	21.6 ± 2.7	24.2 ± 2.4	14.5 ± 2.8	16.9 ± 4.0
2020	17.7 ± 8.5	21.7 ± 2.8	23.8 ± 3.3	16.2 ± 2.5	20.9 ± 3.5

Since the sum of PFSA is so strongly dominated by PFOS that the other substances of this group can almost be neglected. The percentage of PFOS in the PFAA load of the liver samples analyzed from different study areas differed between 85.9% ± 4.3% (Kottenforst in 2020) and 89.5% ± 2.6% (Hesse in 2021). Although the sample numbers differed largely between only three samples from the Eifel region and Rhineland-Palatinate, eight samples from Hesse and 68 samples from the Kottenforst area in 2020, PFOS showed similar percentages for all areas.

## Conclusion

The livers of the wild boars investigated in this study were contaminated with PFAA to a high degree and should not be used as food or as a component of a food due to the associated health hazard. The PFAA levels within the locally studied population were only partially homogeneous. PFOS could be confirmed as a lead substance that was responsible for 85.9% ± 4.1% of the total PFAA contamination across the animals of the studied population. Similar concentrations were also found in smaller comparison groups from different regions in western Germany. The proportion of short-chain PFAA, especially PFCA, varied strongly, which was probably due to their short half-life and high mobility within the organism of wild boar. It was also shown for the first time that wild boar fetuses are contaminated with PFAA through their mothers, since their PFAA concentrations corresponded to those of their dams, especially for short-chain PFCA. The proportion of PFOS, however, was considerably lower in fetuses, which is indicative that the animals accumulate long-chain PFAA over the course of their lives via food. Whether the PFCA transferred by the mother to the fetuses have an effect on fetal development has not yet been clarified. This study also showed a stable relationship between the concentration of PFCA and the sum of PFSA in wild boar liver that is found in different regions. Our results are based on samples from two consecutive years and one enclosed forest area, with comparison groups that consisted of less than five liver samples each. Thus, further research should examine the present findings to determine whether wild boar populations in similar environmental conditions show similar PFAA load patterns regarding the ratios between long-chain PFCA and the dominant share of PFOS.

## Supplementary Information

Below is the link to the electronic supplementary material.

Supplementary file1 (DOCX 48 KB)

## Data Availability

All data generated or analyzed during this study are included in this published article (and its supplementary information files).
