# Supplementary material for: Exposure of a single wild boar population in North Rhine-Westphalia (Germany) to perfluoroalkyl acids
Source: Environ Sci Pollut Res Int. 2022 Sep 28;30(6):15575–84. doi: 10.1007/s11356-022-23086-6 (PMC9908673; doi:10.1007/s11356-022-23086-6)
Supplement: Supplementary file 1 — Supplementary file1 (DOCX 48 KB) [file 11356_2022_23086_MOESM1_ESM.docx]

*Table S1: Information for all animals from the Kottenforst area.*

| animal | BW-Group | sex | year | animal | BW-Group | sex | year |
| --- | --- | --- | --- | --- | --- | --- | --- |
| 2019-01 | >20kg | female | 2019 | 2020-24 | <20kg | female | 2020 |
| 2019-02 | >20kg | male | 2019 | 2020-25 | <20kg | male | 2020 |
| 2019-03 | >20kg | male | 2019 | 2020-26 | <20kg | male | 2020 |
| 2019-04 | >20kg | male | 2019 | 2020-27 | >20kg | female | 2020 |
| 2019-05 | >20kg | male | 2019 | 2020-28 | >20kg | male | 2020 |
| 2019-06 | <20kg | female | 2019 | 2020-29 | >20kg | male | 2020 |
| 2019-07 | >20kg | female | 2019 | 2020-30 | >20kg | female | 2020 |
| 2019-08 | >20kg | male | 2019 | 2020-31 | >20kg | female | 2020 |
| 2019-09 | >20kg | male | 2019 | 2020-32 | >20kg | male | 2020 |
| 2019-10 | <20kg | female | 2019 | 2020-33 | >20kg | male | 2020 |
| 2019-11 | >20kg | male | 2019 | 2020-34 | <20kg | female | 2020 |
| 2019-12 | <20kg | male | 2019 | 2020-35 | <20kg | male | 2020 |
| 2019-13 | <20kg | female | 2019 | 2020-36 | >20kg | female | 2020 |
| 2019-14 | >20kg | female | 2019 | 2020-37 | <20kg | male | 2020 |
| 2019-15 | >20kg | female | 2019 | 2020-38 | >20kg | female | 2020 |
| 2019-16 | >20kg | male | 2019 | 2020-39 | >20kg | male | 2020 |
| 2019-17 | >20kg | female | 2019 | 2020-40 | >20kg | male | 2020 |
| 2019-18 | >20kg | female | 2019 | 2020-41 | >20kg | male | 2020 |
| 2019-19 | >20kg | female | 2019 | 2020-42 | >20kg | female | 2020 |
| 2019-20 | <20kg | female | 2019 | 2020-43 | <20kg | female | 2020 |
| 2019-21 | >20kg | female | 2019 | 2020-44 | >20kg | female | 2020 |
| 2019-22 | >20kg | female | 2019 | 2020-45 | >20kg | female | 2020 |
| 2020-01 | <20kg | female | 2020 | 2020-46 | <20kg | female | 2020 |
| 2020-02 | >20kg | female | 2020 | 2020-47 | >20kg | female | 2020 |
| 2020-03 | <20kg | female | 2020 | 2020-48 | <20kg | male | 2020 |
| 2020-04 | >20kg | male | 2020 | 2020-49 | >20kg | female | 2020 |
| 2020-05 | >20kg | female | 2020 | 2020-50 | >20kg | female | 2020 |
| 2020-06 | <20kg | female | 2020 | 2020-51 | >20kg | female | 2020 |
| 2020-07 | >20kg | male | 2020 | 2020-52 | >20kg | female | 2020 |
| 2020-08 | <20kg | female | 2020 | 2020-53 | <20kg | male | 2020 |
| 2020-09 | >20kg | male | 2020 | 2020-54 | <20kg | male | 2020 |
| 2020-10 | >20kg | female | 2020 | 2020-55 | <20kg | male | 2020 |
| 2020-11 | >20kg | male | 2020 | 2020-56 | <20kg | female | 2020 |
| 2020-12 | >20kg | female | 2020 | 2020-57 | >20kg | female | 2020 |
| 2020-13 | <20kg | female | 2020 | 2020-58 | <20kg | female | 2020 |
| 2020-14 | >20kg | male | 2020 | 2020-59 | >20kg | male | 2020 |
| 2020-15 | >20kg | female | 2020 | 2020-60 | >20kg | female | 2020 |
| 2020-16 | >20kg | female | 2020 | 2020-61 | >20kg | male | 2020 |
| 2020-17 | >20kg | female | 2020 | 2020-62 | >20kg | male | 2020 |
| 2020-18 | >20kg | female | 2020 | 2020-63 | >20kg | female | 2020 |
| 2020-19 | >20kg | female | 2020 | 2020-64 | >20kg | male | 2020 |
| 2020-20 | >20kg | male | 2020 | 2020-65 | <20kg | female | 2020 |
| 2020-21 | >20kg | female | 2020 | 2020-66 | >20kg | female | 2020 |
| 2020-22 | >20kg | male | 2020 | 2020-67 | >20kg | female | 2020 |
| 2020-23 | >20kg | male | 2020 | 2020-68 | >20kg | female | 2020 |

*Table S2: Overview of the liquid chromatography (LC) system and the gradient used to separate the PFAS.*

| **LC-System 1290 Infinity II (Agilent Technologies - Waldbronn, Germany)** | | | |
| --- | --- | --- | --- |
| Column | ZORBAX Eclipse Plus C18 2.1x50mm 1.8 µm  (Agilent Technologies - Waldbronn, Germany) | | |
| MCT temperature | 50°C | | |
| Eluent A | Water:Acetonitrile (98:2) + 20mM CH_3_COONH_4_ | | |
| Eluent B | Methanol + 20mM CH_3_COONH_4_ | | |
| Injection volume | 10 µL | | |
| Flow rate | 0.5 ml/min | | |
| Run time | 15 min | | |
| **Gradient** | **Time [min]** | **Eluent A [%]** | **Eluent B [%]** |
|  | 0.0 | 80 | 20 |
|  | 0.5 | 70 | 30 |
|  | 8.0 | 20 | 80 |
|  | 10.5 | 20 | 80 |
|  | 11.0 | 80 | 20 |

*Table S3: Retention time (RT) and mass transitions of investigated PFAS and internal standards (ISTD)*

| **Compound** | **Precursor [m/z]** | **Product [m/z]** | **RT [min]** | **Compound** | **Precursor [m/z]** | **Product [m/z]** | **RT [min]** |
| --- | --- | --- | --- | --- | --- | --- | --- |
| **PFCA** |  |  |  | **PFSA** |  |  |  |
| PFBA | 213 | 169 | 0.87 | PFBS | 299 | 99 | 2.67 |
| PFPeA | 263 | 219 | 2.47 |  |  | 80 |  |
| PFHxA | 313 | 269 | 3.16 | PFHxS | 399 | 99 | 4.05 |
|  |  | 119 |  |  |  | 80 |  |
| PFHpA | 363 | 319 | 3.92 | PFHpS | 449 | 99 | 4.93 |
|  |  | 169 |  |  |  | 80 |  |
| PFOA | 413 | 369 | 4.85 | PFOS | 499 | 99 | 5.90 |
|  |  | 169 |  |  |  | 80 |  |
| PFNA | 463 | 419 | 5.85 | **ISTD** |  |  |  |
|  |  | 169 |  | 13C4-PFBA | 217 | 172 | 0.86 |
| PFDA | 513 | 469 | 8.87 | 13C2-PFHxA | 315 | 270 | 3.16 |
|  |  | 219 |  | 13C4-PFOA | 417 | 372 | 4.85 |
| PFUnDA | 563 | 519 | 7.78 | 13C5-PFNA | 468 | 423 | 5.86 |
|  |  | 219 |  | 13C2-PFDA | 515 | 470 | 6.83 |
| PFDoDA | 613 | 569 | 8.56 | 13C2-PFUnDA | 565 | 520 | 7.77 |
|  |  | 269 |  | 13C2-PFDoDA | 615 | 570 | 8.61 |
|  |  | 619 |  | 18O2-PFHxS | 403 | 103 | 4.04 |
|  |  |  |  | 13C4-PFOS | 503 | 80 | 5.89 |
|  |  |  |  |  |  |  |  |
|  |  |  |  |  |  |  |  |

*Table S4: Limit of detection and limit of quantification for PFAS analyzed in wild boar liver and muscle tissue.*

| analyte | limit of detection [µg/kg] | limit of quantification [µg/kg] |
| --- | --- | --- |
| PFBA | 0.04 | 0.14 |
| PFPeA | 0.014 | 0.052 |
| PFHxA | 0.002 | 0.008 |
| PFHpA | 0.034 | 0.122 |
| PFOA | 0.024 | 0.086 |
| PFNA | 0.02 | 0.074 |
| PFDA | 0.034 | 0.124 |
| PFUnDA | 0.052 | 0.18 |
| PFDoDA | 0.038 | 0.134 |
| PFBS | 0.014 | 0.054 |
| PFHxS | 0.058 | 0.2 |
| PFHpS | 0.094 | 0.314 |
| PFOS | 0.042 | 0.15 |

*Table S5: Recovery rates for PFAS analyzed in wild boar liver and muscle tissue at two concentration levels.*

| analyte | recovery rate [%] | |
| --- | --- | --- |
|  | 200 ng/L | 1800 ng/L |
| PFBA | 127.0 | 81.6 |
| PFPeA | 117.1 | 86.5 |
| PFHxA | 126.7 | 88.1 |
| PFHpA | 123.1 | 92.6 |
| PFOA | 157.7 | 96.8 |
| PFNA | 122.6 | 95.0 |
| PFDA | 130.1 | 96.1 |
| PFUnDA | 131.4 | 92.5 |
| PFDoDA | 146.3 | 81.3 |
| PFBS | 126.4 | 102.5 |
| PFHxS | 125.7 | 101.5 |
| PFOS | 148.1 | 100.6 |

*Table S6: PFAS Concentrations in wild boar liver in µg/kg, obtained from the Kottenforst study area.*

| animal | PFBA | PFPeA | PFHxA | PFHpA | PFOA | PFNA | PFDA | PFUnDA | PFDoDA | PFBS | PFHxS | PFHpS | PFOS |
| --- | --- | --- | --- | --- | --- | --- | --- | --- | --- | --- | --- | --- | --- |
| 2019-01 | 1.0 | 0.1 | 0.5 | 2.0 | 7.9 | 7.9 | 9.3 | 6.1 | 5.8 | 0.87 | 2.5 | 2.2 | 350 |
| 2019-02 | 0.4 | 0.1 | 1.2 | 6.8 | 32.0 | 17.0 | 15.0 | 6.9 | 7.9 | 1.6 | 3.8 | 4.1 | 490 |
| 2019-03 | 0.3 | 0.1 | 0.5 | 2.6 | 13.0 | 9.3 | 13.0 | 8.7 | 13.0 | 0.99 | 3.7 | 3.2 | 280 |
| 2019-04 | 0.9 | 0.3 | 1.0 | 5.7 | 23.0 | 13.0 | 14.0 | 5.9 | 6.2 | 1.3 | 3.3 | 3.1 | 570 |
| 2019-05 | 1.0 | 0.4 | 1.0 | 3.7 | 17.0 | 14.0 | 16.0 | 9.2 | 13.0 | 1.8 | 4.4 | 4.6 | 520 |
| 2019-06 | 0.7 | 0.1 | 0.2 | 1.8 | 7.7 | 8.4 | 7.5 | 4.7 | 5.1 | 1.3 | 1.6 | 1.5 | 210 |
| 2019-07 | 1.3 | 0.1 | 0.2 | 2.5 | 15.0 | 15.0 | 15.0 | 7.1 | 8.0 | 0.84 | 2.6 | 3.5 | 440 |
| 2019-08 | 1.0 | 0.2 | 0.7 | 3.7 | 17.0 | 14.0 | 15.0 | 9.3 | 16.0 | 1 | 2.7 | 2.9 | 460 |
| 2019-09 | 0.7 | 0.2 | 0.7 | 2.4 | 12.0 | 9.2 | 13.0 | 6.7 | 6.9 | 1.1 | 2.7 | 2.9 | 330 |
| 2019-10 | 0.83 | <LOQ | 0.22 | 1.4 | 7.6 | 7.1 | 7.2 | 4.8 | 5.6 | 0.72 | 1.5 | 1.6 | 220 |
| 2019-11 | 0.57 | 0.33 | 1.8 | 7.4 | 30 | 16 | 20 | 7.6 | 9.4 | 3.3 | 6.8 | 7.9 | 690 |
| 2019-12 | 0.89 | 0.06 | 0.33 | 2.5 | 9.6 | 8.6 | 8.1 | 6 | 7.1 | 0.92 | 2.1 | 1.8 | 210 |
| 2019-13 | 0.93 | 0.06 | 0.32 | 2 | 7.8 | 6.5 | 5.8 | 3.7 | 4.4 | 0.58 | 1.3 | 1.2 | 140 |
| 2019-14 | 0.5 | 0.06 | 0.23 | 1.1 | 7.3 | 11 | 11 | 6.3 | 6.6 | 0.71 | 1.7 | 2.8 | 690 |
| 2019-15 | 0.65 | 0.08 | 0.29 | 1.1 | 4.1 | 6.3 | 8.8 | 6.1 | 6.9 | 0.61 | 1.3 | 1.8 | 310 |
| 2019-16 | 1.4 | 0.06 | 0.2 | 1.6 | 8.3 | 13 | 14 | 8.1 | 9.6 | 0.77 | 2.8 | 3.6 | 390 |
| 2019-17 | 0.66 | 0.05 | 0.16 | 0.85 | 2.9 | 5 | 7 | 5 | 5.9 | 0.54 | 1.1 | 1.4 | 240 |
| 2019-18 | 1.1 | 0.05 | 0.2 | 2.2 | 17 | 15 | 18 | 14 | 17 | 0.99 | 2.3 | 3.8 | 750 |
| 2019-19 | 0.79 | 0.08 | 0.17 | 1.4 | 7.2 | 12 | 11 | 6.4 | 8.3 | 0.71 | 1.8 | 2.8 | 300 |
| 2019-20 | 0.76 | 0.1 | 0.39 | 2.3 | 12 | 11 | 11 | 6.2 | 6.8 | 1 | 1.7 | 1.8 | 240 |
| 2019-21 | 1.1 | 0.14 | 0.37 | 2.4 | 9.2 | 10 | 13 | 8.2 | 10 | 0.69 | 2.5 | 3.8 | 560 |
| 2019-22 | 1.1 | 0.09 | 0.3 | 1.8 | 9.2 | 12 | 14 | 7.7 | 8.3 | 1 | 1.9 | 3.3 | 540 |
| 2020-01 | <LOD | 1.2 | 1.6 | 1.7 | 4.3 | 7.4 | 7.7 | 6.7 | 8.9 | 1.4 | 2.1 | 2.8 | 220 |
| 2020-02 | 1.7 | 1.3 | 1.8 | 2.4 | 8.6 | 8.9 | 11 | 7 | 9.8 | 1.4 | 2.4 | 3.5 | 330 |
| 2020-03 | 2 | 1.5 | 2.1 | 2.6 | 7.1 | 10 | 11 | 8.2 | 11 | 2 | 2.9 | 3.4 | 270 |
| 2020-04 | 1.5 | 1.4 | 2.5 | 4.7 | 16 | 16 | 13 | 9.2 | 11 | 2.9 | 5.1 | 4.6 | 350 |
| 2020-05 | 2.4 | 0.88 | 0.45 | 1.8 | 9.3 | 13 | 16 | 12 | 13 | 1.9 | 6.5 | 6.6 | 890 |
| 2020-06 | 1.6 | 1 | 0.98 | 1.9 | 7.4 | 11 | 16 | 12 | 16 | 1.7 | 3 | 3.7 | 340 |
| 2020-07 | 2.3 | 1.7 | 3 | 3.8 | 13 | 9.9 | 13 | 7 | 10 | 2.7 | 4.9 | 4.9 | 450 |
| 2020-08 | 1.3 | 1 | 1.5 | 1.9 | 5.7 | 7.3 | 7 | 5.2 | 6.9 | 1.2 | 2 | 2.2 | 250 |
| 2020-09 | 1.7 | 1.7 | 2.2 | 4.4 | 17 | 12 | 12 | 6.8 | 7.2 | 3 | 14 | 6.7 | 390 |
| 2020-10 | 1.5 | 1 | 1.2 | 1.9 | 1.3 | 9.8 | 14 | 8.8 | 12 | 1.5 | 2 | 1.8 | 290 |
| 2020-11 | 1.4 | 0.95 | 1.6 | 2.2 | 11 | 11 | 10 | 6.4 | 8.9 | 1.9 | 4.8 | 3.6 | 590 |
| 2020-12 | 1.9 | 1.2 | 0.57 | 1.4 | 4.6 | 7.4 | 8.6 | 5.8 | 7.8 | 1.5 | 3.1 | 2.9 | 310 |
| 2020-13 | 1.9 | 0.98 | 1.1 | 2.7 | 9 | 11 | 15 | 10 | 14 | 1.7 | 2.6 | 2.5 | 400 |
| 2020-14 | 2.1 | 1.2 | 1.6 | 3.1 | 8.4 | 10 | 14 | 9.4 | 12 | 2 | 3 | 2.9 | 470 |
| 2020-15 | 1.8 | 1.2 | 1.6 | 1.7 | 7.1 | 11 | 16 | 11 | 15 | 1.7 | 3.3 | 3.7 | 460 |
| 2020-16 | <LOD | 1.2 | 1.1 | 1.7 | 1.3 | 12 | 14 | 9 | 10 | 1.5 | 4.1 | 4.3 | 510 |
| 2020-17 | 1.8 | 1.1 | 1.2 | 2.4 | 7.9 | 10 | 14 | 8.4 | 12 | 1.6 | 2.4 | 2.6 | 740 |
| 2020-18 | 2.9 | 1.6 | 2.5 | 2.7 | 10 | 9.1 | 13 | 8.1 | 11 | 1.8 | 3.5 | 4.1 | 530 |
| 2020-19 | 1.7 | 1.1 | 1.3 | 2.4 | 1.2 | 9.9 | 13 | 8.5 | 9.9 | 1.7 | 3.2 | 2.4 | 580 |
| 2020-20 | 1.6 | 1.2 | 1.7 | 1.4 | 5.1 | 10 | 9.1 | 5.9 | 6.8 | 1.4 | 2.6 | 3.3 | 320 |
| 2020-21 | 1.6 | 1.3 | 1.7 | 1.4 | 4.6 | 8.5 | 8.6 | 6.7 | 8.5 | 1.3 | 2.8 | 2.9 | 140 |
| 2020-22 | 1.3 | 1.1 | 1.9 | 5.5 | 22 | 12 | 11 | 5.8 | 6.5 | 2.5 | 11 | 6.8 | 260 |
| 2020-23 | 2.7 | 1.4 | 2.2 | 2.6 | 8.5 | 11 | 14 | 8.2 | 10 | 1.9 | 3.1 | 4.2 | 540 |
| 2020-24 | <LOD | 1.2 | 1.3 | 3.7 | 1.4 | 13 | 12 | 8.6 | 12 | 2.4 | 4.5 | 3.1 | 290 |
| 2020-25 | 2.9 | 0.74 | 1.7 | 2.8 | 11 | 11 | 12 | 9 | 11 | 2.8 | 3.7 | 3.7 | 400 |
| 2020-26 | 3.2 | 1.5 | 3 | 3.3 | 1.5 | 13 | 14 | 12 | 16 | 3.8 | 5.1 | 4.5 | 670 |
| 2020-27 | <LOD | 0.83 | 1.5 | 3.4 | 2.2 | 9.8 | 11 | 8.3 | 9.6 | 2.2 | 2.7 | 2.4 | 690 |
| 2020-28 | <LOD | 1.2 | 1.8 | 4.2 | 2.2 | 14 | 20 | 9.8 | 15 | 1.8 | 5.5 | 5.7 | 720 |
| 2020-29 | 2.3 | 1.5 | 2 | 3.3 | 9.1 | 7.8 | 11 | 7.5 | 11 | 2 | 3.8 | 2.9 | 430 |
| 2020-30 | <LOD | 1.1 | 1.1 | 1.7 | 1.4 | 10 | 8.6 | 6.1 | 6.8 | 1.4 | 4.2 | 2.2 | 360 |
| 2020-31 | 1.8 | 1.2 | 1.8 | 2.7 | 11 | 9.3 | 9.9 | 8.4 | 10 | 2 | 3.1 | 3.1 | 460 |
| 2020-32 | 2.1 | 1.1 | 1.6 | 1.7 | 8 | 7 | 6.1 | 4.6 | 6.1 | 1.7 | 2.3 | 3 | 290 |
| 2020-33 | 0.78 | 0.82 | 1.6 | 5.2 | 25 | 12 | 11 | 5.5 | 6.7 | 2 | 7.5 | 5 | 450 |
| 2020-34 | 2.1 | 0.95 | 1.5 | 2.6 | 10 | 9.8 | 9.4 | 6.9 | 8.9 | 1.8 | 3.7 | 3.2 | 360 |
| 2020-35 | 1.6 | 0.82 | 1.4 | 2.6 | 8.5 | 10 | 10 | 7.2 | 10 | 2 | 2.8 | 4.2 | 360 |
| 2020-36 | 1.4 | 1.2 | 1.3 | 2.1 | 7 | 9.8 | 11 | 6.7 | 7.8 | 1.4 | 2.5 | 2.6 | 290 |
| 2020-37 | 1.6 | 0.83 | 1.2 | 3.1 | 9.9 | 15 | 14 | 10 | 14 | 2.4 | 3.7 | 3 | 450 |
| 2020-38 | 1.4 | 0.96 | 1.4 | 2.7 | 10 | 8.5 | 7.9 | 5.7 | 7.4 | 2.2 | 3.5 | 2.4 | 300 |
| 2020-39 | 3 | 1.8 | 1.9 | 4.7 | 16 | 17 | 18 | 14 | 20 | 2.8 | 4.3 | 4.3 | 530 |
| 2020-40 | 2.4 | 1.2 | 1.8 | 2.9 | 8.5 | 8.4 | 8.6 | 6.8 | 8.7 | 2.2 | 3.7 | 2.3 | 400 |
| 2020-41 | 2.8 | 1.2 | 1.5 | 3.3 | 7.2 | 8.1 | 12 | 7.2 | 9.8 | 1.7 | 3 | 2.1 | 370 |
| 2020-42 | 1.6 | 1.3 | 1.2 | 1.6 | 3.7 | 6.9 | 8.8 | 6.9 | 7.6 | 1.7 | 2 | 2 | 330 |
| 2020-43 | 3.7 | 1.4 | 1.7 | 3.8 | 11 | 11 | 10 | 8.1 | 9.4 | 2.4 | 3.3 | 2.7 | 260 |
| 2020-44 | 1.8 | 0.56 | 1.3 | 2.4 | 7.1 | 8.2 | 11 | 7.9 | 9.1 | 1.4 | 2.6 | 2.7 | 320 |
| 2020-45 | 1.9 | 1.3 | 2 | 2 | 6.8 | 11 | 10 | 5.7 | 7.1 | 1.4 | 3.2 | 3.5 | 830 |
| 2020-46 | 2.3 | 1.4 | 2.1 | 3.5 | 10 | 12 | 11 | 8 | 10 | 2.1 | 4.1 | 3.8 | 370 |
| 2020-47 | 1.4 | 1.4 | 1.7 | 0.73 | <LOD | <LOD | <LOD | <LOD | <LOD | <LOD | 1.5 | <LOD | 350 |
| 2020-48 | 2.1 | 1.5 | 2.2 | 2.1 | 5.2 | 7.5 | 7.4 | 5.4 | 6.7 | 1.5 | 2.4 | 2.7 | 240 |
| 2020-49 | 2.3 | 1.3 | 1.5 | 2.2 | 1.1 | 7.8 | 9.2 | 6.3 | 6.4 | 1.8 | 2.5 | 2.8 | 400 |
| 2020-50 | 2.1 | 1.4 | 2.2 | 3 | 18 | 11 | 9.4 | 7.5 | 9.3 | 1.8 | 3.5 | 3.8 | 250 |
| 2020-51 | 1.6 | 1 | 1.6 | 1.6 | 5.7 | 6.5 | 11 | 4.9 | 7.6 | 1.2 | 2.1 | 3.3 | 630 |
| 2020-52 | 2.3 | 1.3 | 1.9 | 2.8 | 13 | 12 | 14 | 10 | 12 | 2.9 | 6.1 | 5.6 | 580 |
| 2020-53 | 2.1 | 1.4 | 1.6 | 4.1 | 15 | 12 | 11 | 7.7 | 10 | 2.7 | 3.6 | 3.6 | 310 |
| 2020-54 | <LOD | <LOD | <LOD | 4.2 | 8.9 | 10 | 9.8 | 5.9 | 8.4 | 2 | 3.6 | 3.5 | 230 |
| 2020-55 | <LOD | <LOD | <LOD | 3.6 | 9.7 | 8.9 | 8.2 | 5.7 | 7.8 | 2 | 3 | 2.9 | 220 |
| 2020-56 | 1.6 | 1 | 1.4 | 2.6 | 10 | 11 | 9.7 | 6.6 | 9 | 1.9 | 3.5 | 3.2 | 310 |
| 2020-57 | 1.7 | 0.89 | 1.1 | 2.4 | 11 | 8.7 | 9.7 | 7 | 7.9 | 1.9 | 4.3 | 4 | 570 |
| 2020-58 | 2.1 | 1.1 | 1.3 | 1.9 | 5.7 | 6.9 | 9.8 | 5.2 | 8.4 | 1.4 | 2.6 | 3.3 | 430 |
| 2020-59 | 3.8 | 1.5 | 1.8 | 4.7 | 25 | 16 | 13 | 9.3 | 11 | 2.2 | 3.5 | 3.4 | 450 |
| 2020-60 | 1.3 | 1 | 1.3 | 2.6 | 12 | 13 | 13 | 7.1 | 7.2 | 1.5 | 3 | 2.6 | 370 |
| 2020-61 | 1.3 | 1.1 | 1.8 | 4.3 | 16 | 12 | 13 | 11 | 15 | 2.1 | 4.3 | 4.3 | 630 |
| 2020-62 | 1.5 | 1.3 | 1.7 | 4.9 | 15 | 10 | 9.9 | 6.5 | 8.6 | 2.4 | 4.1 | 3.9 | 360 |
| 2020-63 | 1.4 | 0.91 | 1 | 1.7 | 6.9 | 7.4 | 7.2 | 4.2 | 4.5 | 1.1 | 2.6 | 3.1 | 350 |
| 2020-64 | 2.3 | 1.7 | 2.1 | 4.3 | 28 | 21 | 17 | 10 | 12 | 2.4 | 4.2 | 5.2 | 530 |
| 2020-65 | 1.6 | 1.5 | 1.9 | 2.4 | 7.9 | 16 | 17 | 11 | 15 | 1.9 | 3.3 | 3.6 | 420 |
| 2020-66 | 2.4 | 1.6 | 1.6 | 2.6 | 12 | 20 | 20 | 13 | 14 | 2 | 4.4 | 4.5 | 740 |
| 2020-67 | 1.5 | 0.89 | 1.4 | 2.6 | 10 | 14 | 17 | 12 | 16 | 1.6 | 4.6 | 4 | 760 |
| 2020-68 | 1.4 | 0.88 | 1.3 | 1.5 | 6.5 | 6.3 | 6.8 | 4.3 | 5.1 | 0.64 | 3.7 | 3 | 370 |

*Table S7: PFAS Concentrations in wild boar liver in µg/kg, obtained from the different study areas.*

| animal | PFBA | PFPeA | PFHxA | PFHpA | PFOA | PFNA | PFDA | PFUnDA | PFDoDA | PFBS | PFHxS | PFHpS | PFOS |
| --- | --- | --- | --- | --- | --- | --- | --- | --- | --- | --- | --- | --- | --- |
| Eifel-01 | 3,6 | 2,3 | 2,6 | 3,1 | 8,6 | 12 | 11 | 8,1 | 9,0 | 2,1 | 3,0 | 4,0 | 280 |
| Eifel-02 | 2,7 | 1,6 | 2,2 | 2,1 | 4,1 | 9,7 | 10 | 7,7 | 9,8 | 1,7 | 4,1 | 3,0 | 340 |
| Eifel-03 | 4,0 | 3,0 | 2,8 | 2,9 | 4,8 | 7,9 | 9,7 | 6,9 | 8,1 | 2,1 | 2,5 | 2,3 | 480 |
| RLP-01 | 5,9 | 2,3 | 2,2 | 3,5 | 7,2 | 15 | 17 | 12 | 16 | 1,9 | 2,7 | 3,9 | 570 |
| RLP-02 | 4,6 | 2,3 | 2,4 | 3,7 | 6,4 | 18 | 21 | 12 | 18 | 1,9 | 2,0 | 3,6 | 800 |
| RLP-03 | 3,8 | 2,0 | 2,8 | 4,1 | 5,6 | 11 | 13 | 12 | 15 | 2,2 | 1,8 | 3,4 | 400 |
| Hessen-01 | 3,7 | 4,2 | 2,4 | 2,7 | 5,2 | 4,4 | 3,9 | 3,4 | 3,9 | 1,2 | 9,1 | <LOD | 160 |
| Hessen-02 | <LOD | 1,1 | 1,4 | 1,9 | 2,9 | 3,5 | 3,4 | 2,9 | 2,6 | 0,92 | 9,4 | <LOD | 200 |
| Hessen-03 | <LOD | 1,3 | 1,4 | 2,3 | 4,2 | 6,1 | 4,7 | 3,4 | 3,4 | 1,0 | 12 | 2,0 | 250 |
| Hessen-04 | 0,18 | 1,3 | 1,3 | 2,9 | 8,6 | 12 | 11 | 7,0 | 7,0 | 1,3 | 20 | 4,0 | 530 |
| Hessen-05 | <LOD | 1,2 | 1,6 | 2,0 | 4,5 | 6,9 | 5,5 | 3,1 | 3,4 | 1,1 | 7,7 | 1,4 | 260 |
| Hessen-06 | <LOD | 1,0 | 1,4 | 2,0 | 3,8 | 5,6 | 5,1 | 4,4 | 4,3 | 1,0 | 8,8 | 1,5 | 200 |
| Hessen-07 | 0,46 | 2,2 | 1,8 | 3,2 | 8,0 | 6,4 | 5,5 | 3,6 | 4,3 | 1,1 | 17 | 2,5 | 290 |
| Hessen-08 | 0,46 | 1,1 | 1,3 | 2,5 | 5,5 | 7,3 | 5,9 | 4,4 | 4,6 | 0,9 | 14 | 2,2 | 250 |

*Table S8: PFOS concentration in wild boar muscle tissue in µg/kg.*

| animal | PFOS | animal | PFOS |
| --- | --- | --- | --- |
| 2020-01 | 3.7 | 2020-35 | 3.9 |
| 2020-02 | 6.8 | 2020-36 | 11 |
| 2020-03 | <LOD | 2020-38 | 9.8 |
| 2020-04 | 3.2 | 2020-39 | 6.9 |
| 2020-05 | 8.1 | 2020-40 | 9.8 |
| 2020-06 | 21 | 2020-41 | 10 |
| 2020-07 | 5.3 | 2020-42 | 8.7 |
| 2020-08 | 3.4 | 2020-43 | 8.8 |
| 2020-10 | 7.3 | 2020-44 | 5 |
| 2020-11 | 8.8 | 2020-45 | 7.5 |
| 2020-12 | 5.5 | 2020-46 | 3.3 |
| 2020-13 | 13 | 2020-47 | 7.1 |
| 2020-14 | 9.5 | 2020-48 | 2.9 |
| 2020-15 | 4.4 | 2020-49 | 7.3 |
| 2020-16 | 3.7 | 2020-50 | 5.2 |
| 2020-17 | 13 | 2020-51 | 5.4 |
| 2020-18 | 6.5 | 2020-52 | 8.1 |
| 2020-19 | 6.3 | 2020-54 | <LOD |
| 2020-20 | 5.1 | 2020-57 | 12 |
| 2020-21 | 11 | 2020-58 | 11 |
| 2020-22 | 8.7 | 2020-59 | <LOD |
| 2020-23 | 5.4 | 2020-60 | 8.3 |
| 2020-25 | 10 | 2020-62 | 6.6 |
| 2020-27 | 9.3 | 2020-63 | 15 |
| 2020-28 | 14 | 2020-64 | 8.9 |
| 2020-29 | 9.9 | 2020-65 | 11 |
| 2020-30 | 16 | 2020-66 | 12 |
| 2020-31 | 7.6 | 2020-68 | 9.6 |
| 2020-33 | 7.4 |  |  |
